# Editorial: Transformational competences and the Fourth Industrial Revolution

**DOI:** 10.3389/fpsyg.2024.1504360

**Published:** 2024-12-16

**Authors:** Claude-Hélène Mayer, Rudolf M. Oosthuizen, Elisabeth Vanderheiden

**Affiliations:** ^1^Department of Industrial Psychology and People Management, College of Business and Economics, University of Johannesburg, Johannesburg, South Africa; ^2^Department of Industrial and Organisational Psychology, University of South Africa, Pretoria, South Africa; ^3^Global Institute for Transcultural Research, Römerberg, Germany

**Keywords:** 4IR competencies, transformation, future of work, competence development, Fourth Industrial Revolution “industry 4.0”

The move toward the Fourth Industrial Revolution (4IR) is rapidly progressing and organizations are changing faster than ever before (Bughin et al., 2018; SAICA, 2023). It has been predicted that the workforce of machines will complete more jobs than humans in 2025 (Smith and Anderson, 2014). Others highlight that through the 4IR and especially through AI and robotics, many more jobs will be created (Choudhury, 2018). The 4IR will be driven by AI, System Dynamics, Human-Machine and Machine-Machine interaction (Mayer and Oosthuizen, 2020a).

New AI applications, such as chatGBT, LaMDA or BARD are leading to far-reaching changes in all learning and work contexts and require far-reaching realignments in terms of competences and skills (Kroon et al., 2021). Adoption and adjustment are critical aspects in dealing with the technological changes and the new world of work (Davies, 2022) and leaders need to focus on developing comprehensibility, manageability, and meaningfulness to cope with the radical changes and promote mental health and wellbeing in organizations of the 4IR (Mayer and Oosthuizen, 2020b).

Many professions and organizations have to change the way the work and how work is allocated (Schwab, 2016; Manyika et al., 2017; Rotatori et al., 2021). One the one hand, the new technologies present possible opportunities and on the other hand they pose challenges and difficulties for employees and organizations in terms of skill development and adaptability (Malomane et al., 2022; Prisecaru, 2016; Schwab, 2017; Xu et al., 2018).

Organizations and workspaces need to collaborate with the educational sector—and in particular higher education—to define skills and competences which individuals across cultures and in specific cultural contexts need to develop to thrive in the 4IR (Rychen, 2016). Mayer (2020) has highlighted that a globalized 4IR workforce needs to develop key concepts, such as creativity, positive emotions, solution-focus and meaning-making in the 4IR to be sustainable and successful. The author further points out that these key concepts need to be developed in educational institutions and required by leaders of the 4IR, as precious skills.

According to the Organization for Economic Cooperation and Development (2023), there is a need to develop the following three transformational skills to shape a desired future: Creating new values, balancing tensions, and dilemmas, and taking responsibility. To create new values, individuals need to ask questions, collaborate with others and try to think outside the box to find innovative solutions (Bentley, 2017; Grayling, 2017). This links a sense of purpose with critical thinking and creativity. In an interdependent world, individuals need to be able to balance conflicting or seemingly incompatible thinking and demands and become comfortable with complexity and ambiguity, taking responsibility for their actions and have a strong moral compass for critical reflection, cooperation with others, and respect for the environment and humanity (Melville and Robert, 2020).

The discourse surrounding the 4IR indeed often presents it as a global phenomenon, characteried by the convergence of digital, physical, and biological technologies and their impact on various aspects of society, economy, and culture. Berger and Frey (2015), as well as Schwab (2016), have contributed significantly to this narrative, emphasizing the widespread nature of technological advancements and their transformative effects on industries and societies worldwide. However, it's essential to acknowledge the perspective that views the 4IR through a cultural lens. Oosthuizen and Mayer (2019) highlight that while the technological advancements associated with the 4IR may have global reach, their adoption, adaptation, and impact can vary significantly across different cultural contexts. Cultural factors such as values, norms, traditions, and governance structures can shape how societies engage with and are affected by these technological changes. This perspective underscores the importance of considering local contexts and cultural nuances when discussing the 4IR and its implications. It suggests that while there may be overarching trends and developments at the global level, the way these manifest and are experienced can differ significantly from one culture to another. This recognition prompts a more nuanced understanding of the 4IR and the need for tailored approaches to address its opportunities and challenges in different cultural settings.

The aim of this *Frontiers Research Topic* is to bring researchers of different disciplines and cultures together to describe, explore and investigate the concepts of the 4IR with specific regard to the question of what transformational skills and competences are needed to thrive within the 4IR scenario. The articles of this Research Topic do not only focus on the workspace and the needed skills and competences, but also on the educational sector. In this context, the question is posed how education can support the development of transformational skills and competences, and what skills and competences are actually needed in with organizational, work, and cultural context.

The Research Topic contains five articles which focus on the 4IR, with digital competences, automation, and artificial intelligence. In the following section, the articles in this *Research Topic* are briefly introduced.

Singaram et al. explore in their qualitative study “*Leading higher education into the Fourth Industrial Revolution: an empirical investigation*” how leaders within higher education adjust their visions, the management and the organsiational structures toward new requirements of learning and skill development. They present findings of a specific higher education institution (HEI) in South Africa and how this institution adjusts to the rapid changes and new demands of skill development in the South African context. Challenges and opportunities are discussed for HEIs with special regard to how leadership may manage the times of transitions toward a more technologised learning environment, and new skill sets in the 4IR.

The second article in this Research Topic is about “*Self-Assessed Digital Competences of Romanian Teachers During the COVID-19 Pandemic*,” authored by Hatos et al.. The authors highlight that during the Pandemic, teachers have been forced to find online teaching methods and where thrown back onto their personal digital skills and abilities to adapt to the radical changes. In their study, they analyse the perceived digical competences of Romanian pre-tertiary cycle teachers, using data from 3,419 self-completed questionnaires. They show differences in teachers of all specialties, gender and professional status, correlating with perceived office digital skills. Finally, the authors present implications for future research, as well as for educational interventions and policies in Romania.

The authors Eich et al. point out in their article “*How automation level influences moral decisions of humans collaborating with industrial robots in different scenarios*” how digitalisation in intelligent manufacturing leads to the development of Industry 4.0/5.0 and human-cyber-physical systems. They highlight that for designing industrial robots in a human-centered way, psychological knowledge concerning judgment and decision-making needs to be gained and integrated. Their article reports results from an experimental study using eight moral dilemmas framed in the context of human-robot-collaboration to examine the influence of spatial distance of an industrial robot and humans on moral decisions. Their results show a large effect of the proximity of the cooperation between robots and humans.

The fourth article in this Research Topic is presented by Singh and Pandey and is titled: “*Artificial intelligence adoption in extended HR ecosystems: enablers and barriers. An abductive case research*.” They point out that their study investigates HRM specific factors that enable and inhibit the adoption of AI in extended HR ecosystems. Methodologically, they use a qualitative case research design with an abductive approach to study three well-known Indian companies at different stages of AI adoption in HR functions. Further, the authors investigate key enablers and barriers to adoption.

Finally, Brands et al. present their findings on the perception of the 4IR in Chartered Accountants. The authors used a qualitative research methodology, collecting data through 14 semi-structured interviews. The findings reveal that influences of the 4IR are radically changing in the Chartered Accountants profession. The new 4IR changes lead to strengthened analytical and advising roles and an increased learning to improve critical thinking, analytical ability, and IT skills. Younger Chartered Accountants, who are more tech-savvy, adapt faster to the digital revolution, while older generations struggle to bridge the technology gap. Additionally, they have concerns about job displacement, while striving for professional advancement and growth.

The editors wish all of the readers of this Research Topic an interesting read and hope to stimulate contemporary and future discourses on the 4IR!
